# SARS-CoV-2 quantitative real time PCR and viral loads analysis among asymptomatic and symptomatic patients: an observational study on an outbreak in two nursing facilities in Campania Region (Southern Italy)

**DOI:** 10.1186/s13027-021-00388-x

**Published:** 2021-06-22

**Authors:** Lorena Cardillo, Claudio de Martinis, Maurizio Viscardi, Claudia Esposito, Emanuela Sannino, Gabriella Lucibelli, Antonio Limone, Stefania Pellino, Rosa Anastasio, Roberta Pellicanò, Loredana Baldi, Giorgio Galiero, Giovanna Fusco

**Affiliations:** 1 Department of Animal Health, Unit of Virology, Istituto Zooprofilattico Sperimentale del Mezzogiorno, Via Salute, 2, 8055 Portici, Naples, Italy; 2 Department of Animal Health, Unit of Applied Biotechnologies and Bioinformatics, Istituto Zooprofilattico Sperimentale del Mezzogiorno, Portici, Naples, Italy; 3 Department of Animal Health, Unit of Biomolecular Diagnostics, Istituto Zooprofilattico Sperimentale del Mezzogiorno, Portici, Naples, Italy; 4 Istituto Zooprofilattico Sperimentale del Mezzogiorno, Portici, Naples, Italy; 5 Department of Prevention, Azienda Sanitaria Locale (ASL) Benevento, Benevento, Italy; 6 Residential and Nursing Home Madonna dell’Arco, Sant’Anastasia, Naples, Italy; 7 Department of Epidemiology and Biostatistic Regional Observatory (OREB), Istituto Zooprofilattico Sperimentale del Mezzogiorno, Portici, Naples, Italy

**Keywords:** SARS-CoV-2, COVID-19, Viral loads, Symptomatic, Asymptomatic, Age, Sex, Risk factors

## Abstract

**Background:**

In December 2019 an outbreak of Severe Acute Respiratory Syndrome Coronavirus 2 was first observed in Wuhan, China. The virus has spread rapidly throughout the world creating a pandemic scenario. Several risk factors have been identified, such as age, sex, concomitant diseases as well as viral load. A key point is the role of asymptomatic people in spreading SARS-CoV-2. An observational study in Southern Italy was conducted in order to elucidate the possible role of asymptomatic individuals related to their viral loads in the transmission of the virus within two nursing facilities.

**Methods:**

Oro-nasopharyngeal swabs from 179 nursing health care workers and patients were collected. SARS-CoV-2 RT-qPCR was performed and viral loads were calculated by using standard curve. A statistical correlation between *viral loads*, the *presence/absence of symptoms*, *age* and *sex* variables was investigated.

**Results:**

SARS-CoV-2 was confirmed in the 50.8 % (*n* = 91) of the cases. Median age of positive individuals resulted higher than negative ones. Over 65 year as well as female individuals showed higher susceptibility to SARS-CoV-2 infection, OR = 3.93 and 2.86, respectively. Among 91 tested positive, the 70.3 % was symptomatic while the 29.7 % was asymptomatic. Median viral loads of asymptomatic individuals were found statistically significant higher than symptomatic ones (*p* = 0.001), while no influence was observed in *age* and *sex* variables. The presence of comorbidities was 8.9 folds higher in patients who showed and developed symptoms compared to non-symptomatic ones. Moreover, higher viral loads were found in patients who remained asymptomatic than pre-symptomatic (*p* = 0.022).

**Conclusions:**

A range from 9.2 to 69 % of confirmed SARS-CoV-2 cases remains asymptomatic, moreover, sporadic transmissions from asymptomatic people are reported, that makes their involvement an important issue to take into account in the spreading control of the virus. An asymptomatic clinical course was observed in the 29.7 % of positive individuals, moreover, median viral loads resulted to be statistically significant when compared to symptomatic ones. Surely, such a relevant frequency should not be ignored in relation to the spread of the disease in an environment which has not only important intrinsic (age, sex, concomitant diseases) but also extrinsic factors such as high population density and close contacts.

**Supplementary Information:**

The online version contains supplementary material available at 10.1186/s13027-021-00388-x.

## Background

On late December 2019, a novel human *Betacoronavirus* caused a cluster of severe acute respiratory syndrome (SARS) cases in Wuhan (China). The virus was later named SARS-CoV-2 by the International Committee of Taxonomy [1]. The global spread of the virus has created a pandemic scenario with over 67 million reported cases and 1.5 million deaths worldwide since the start of the pandemic [2]. On January 2020, Italy was detected as the first country where SARS-CoV-2 shifted away from its origins [3], causing 1.7 million cases, with a 48 years median age, and 58.8 thousand deaths [4].

The disease clinical course is characterized by several factors, from asymptomatic to mild or severe viral respiratory tract infections, up to systemic inflammation and thrombosis [5]. Several other risk factors have also been identified to affect the disease course, such as sex, concomitant diseases, close contact and age [5, 6]. Indeed, European Centre for Disease Control and Prevention (ECDC) has identified 65 years old and older people at a greater risk of hospitalization and death for COVID-19, from 90x higher risk of death for 65–74 years old, up to 630x higher for over 85 years old people [7]. Moreover, Pujadas and colleagues showed that high viral load was correlated to higher mortality rates, suggesting to use quantitative analyses for patient risk-stratification [8], thus, high viral load could act a key role in the severity of COVID-19. These findings were supported by other studies reporting an association between high viral loads and more severe symptoms [6–12]. Otherwise, other studies did not show any statistically significant difference in viral loads between symptomatic and asymptomatic individuals [8, 11–19], while Hasanoglu and colleagues showed that higher viral loads were found in asymptomatic patients when compared to symptomatic ones and that, the more the clinical course of the diseases was severe, the lower viral loads were observed, together with a significant negative trend with increasing age [5]. Thus, the clinical relevance of the role of viral loads is still unclear. The aim of this study was to elucidate the dilemma of the viral loads correlation to symptoms, age and sex, for this reason, an observational study was conducted in a SARS-CoV-2 outbreak in two nursing facilities in Campania region, Southern Italy.

## Methods

### Subjects and data collection

An observational study was carried out by the Istituto Zooprofilattico Sperimentale del Mezzogiorno (IZSM) of Portici, Naples (Southern Italy), as part of routine diagnostic activities of COVID-19, aimed to verify the correlation of viral loads to symptoms, age and sex. Combined oro-nasopharyngeal swabs were performed by the Local Health Authorities. Swabs were transferred to the IZSM in Universal Viral Transport Medium (UTM) (Copan, Brescia, Italy), accompanied by an application form that included records, signalling, symptoms and main health state of each tested person. Next, further data on the follow up of the tested positive individuals were kindly provided by the chief medical officers of the two facilities.

### Nucleic acid extraction and molecular analysis

Nucleic acid extraction was conducted as follows: in Biosafety Level 3 (BLS-3) laboratories, aliquots of 200 µL of UTM were collected from each specimen and submitted to extraction and purification using QIAsymphony DSP Virus/Pathogen Mini Kit (Qiagen, Hilden, Germany) and performed by QIAsymphony automated system (Qiagen) following manufacturer’s instructions, eluted in 60 µL and stored at − 80 °C until use.

Notably, SARS-CoV-2 RT-qPCR was performed in BLS-2,using TaqPath COVID-19 CE-IVD RT-PCR Kit (Thermo Fisher Scientific), approved by the World Health Organization (WHO) [20], that simultaneously amplifies 3 viral targets, ORF1ab gene (FAM), N protein (VIC), S protein (ABY) and MS2 phage (JUN) as internal control. Amplification was carried out in a final volume of 25 µL and included 5 µL of template, TaqPath 1-Step Multiplex Master Mix (4X), probes and specific primer sets for the different SARS-CoV-2 genomic regions. Thermal cycling conditions included an initial Uracil-DNA glycosylase (UNG) incubation step at 25 °C for 2 min, followed by reverse transcription at 53 °C for 10 min, initial denaturation/enzyme activation at 95 °C for 2 min, 40 cycles of denaturation at 95 °C for 3 s and annealing/extension at 60 °C for 30 s. The RT-qPCR was performed on 7500 Fast Real-Time PCR system (Applied Biosystems, Foster City, USA). Viral loads were calculated by using standard curve, as described elsewhere [8]. To generate the real-time PCR standard curve, appropriate 10-fold serial dilutions of the titrated positive control provided by the RT-PCR Kit were performed in triplicate. A standard curve was obtained by linear regression analysis of the threshold cycle (C_t_) value (y-axis) *versus* the log of the initial copy number present in each sample dilution (x-axis). PCR efficiency (E) was calculated as E = 10 (1/slope)^−1^.

### Statistical analysis

Univariate models were first used. The variables considered were: *results* (positive/negative) *age* (</> 65 years) and *sex* (male/female).Prevalence was calculated at a 95 % confidence level. A chi-squared test of association was used to obtain the statistical significance level between groups. A correlation within results and age class was performed by using the Kolmogorov-Smirnov normality test in order to assess the statistical distribution of these two variables. Next, the non-parametric Mann Whitney U test was performed to verify the difference of the median age values in the positive/negative group was statistically significant. For positive results, a correlation between *viral loads* (copies/mL), the presence/absence of symptoms (*symptomatic/asymptomatic*), *age* and *sex* were investigated. For these variables, Kolmogorov-Smirnov and Mann Whitney U test, were performed. Finally, for the evaluation of viral loads among asymptomatic and pre-symptomatic groups, Mann Whitney U test was used, too.

All statistical analyses were performed by using SPSS software, version 24.0 (IBM Corporation). Results were considered statistically significant with a *p* value < 0.05.

## Results

On late March 2020, during lockdown, in a nursing facility for elderly people in Naples province and in a rehabilitation facility, located in Benevento province, Campania, region (Southern Italy), suspected onset of COVID-19 symptoms in some patients were reported. A total of 179 combined oro-nasopharyngeal swabs were performed by the Local Health Authorities on patients and health care workers. Swabs were conducted to the IZSM for diagnostic procedures and SARS-CoV-2 was confirmed in 91 patients (50.8 %).

Patient age ranged between 22 and 98 years, with a median of 65 years. They were divided into two categories: “*age*”, under and over 65 years old, that represented the 48 % (*n* = 86) and 52 % (*n* = 93), respectively, and “*sex*”, male and female, 40.8 % (*n *= 73) and 59.2 % (*n* = 106), respectively. Results are reported in Table 1. A statistically significant difference between over and under 65 years old individuals was observed (*p* < 0.001) and higher susceptibility to SARS-CoV-2 infection was found for elderly people (OR = 3.93). *Sex* variable showed a statistically significant difference (*p* < 0.001) too, and female were found 2.86 folds more susceptible to COVID-19 than male.

**Table 1:** Sex and age statistical analysis

Variable	Total	Positive		95% CI	X^**2**^	***p*** value	OR	OR 95% CI
		n	%					
**Overall**	179	91	50.8	44.04-57.56				
**>65 y**	93	62	66.6	57.02-76.18	**19.405**	**<0.001**	**3.93**	2.11-7.31
**<65 y**	86	29	36.7	26.52-46.88				
**Female**	106	65	61.3	52.03-70.57	**11.43**	**<0.001**	**2.86**	1.54-5.31
**Male**	73	26	35.6	24.62-46.58				

Data analyses concerning *age*, showed that average and median age of tested negative patients (57 years) were lower than positive ones (75 years) (Table 2). Kolmogorov-Smirnov normality test was performed and it demonstrated that *age* variable was not normally distributed (*p* = 0.001), next, Mann Whitney U test verified that the difference between median values of age registered both in positive and negative groups was statistically significant (*p* = 0.001).

**Table 2:** Average and median age evaluation of negative and positive individuals

		AGE					
	Total (n)	Mean	Median	Min	Max	SD	95% CI mean age
**Negative**	88	59	57	22	98	20	55 - 64
**Positive**	91	70	75	24	96	18	66 - 74

The presence/absence of symptoms was evaluated for tested positive. Among 91 individuals, 27 were asymptomatic (29.7 %) at swab sampling time while 64 were symptomatic (70.3 %). The most common symptoms reported were fever (*n* = 32; 35.1 %), dyspnea (*n* = 20; 21.9 %), cough (*n* = 12; 13.2 %), asthenia (*n* = 7; 7.7 %) and gastroenteritis (*n* = 7; 7.7 %), while for six patients clinical data were not available. Viral loads of the samples were analyzed. Viral loads on tested positive individuals ranged from 699 to 4.71 × 10^8^ copies per mL, with a median of 1.46 × 10^5^. Asymptomatic group showed higher median viral load values (1.14 × 10^7^) than symptomatic one (3.39 × 10^4^). Kolmogorov-Smirnov test showed that the *viral load* variable was not normally distributed (*p* = 0.001) followed by the non-parametric Mann Whitney U test performed, thus median viral load values were evaluated and a statistically significant difference was found, too (*p* = 0.001). On the other hand, viral load was not influenced by *age* and *sex* variables. Results are reported in Table 3.

**Table 3:** Evaluation of viral loads of tested positive individuals

Variable	Total (n)	Viral loads (copies/mL)					
		Mean	95% CI	Median	Min	Max	SD
**Overall**	91	2.52E+07	1.05E+07 – 3.99E+07	1.46E+05	6.99E+02	4.71E+08	7.01E+07
**Asymptomatic**	27	3.99E+07	1.20E+07 – 6.77E+07	**1.14E+07**	1.30E+04	2.67E+08	7.03E+07
**Symptomatic**	64	1.90E+07	1.47E+06 – 3.65E+07	**3.39E+04**	6.99E+02	4.71E+08	7.02E+07
**> 65 y**	62	3.09E+07	1.05E+07 – 5.13E+07	1.53E+05	7.17E+02	4.71E+08	8.02E+07
**< 65y**	29	1.85E+07	4.32E+05 – 3.66E+07	1.38E+05	6.99E+02	1.91E+08	4.75E+07
**Female**	65	2.34E+07	5.66E+06 – 4.11E+07	6.17E+04	6.99E+02	4.71E+08	7.16E+07
**Male**	26	2.96E+07	2.94E+06 – 4.63E+07	4.44E+05	1.34E+03	2.46E+08	6.60E+07

Further clinical information on the tested positive patients were obtained. Among asymptomatic patients, 7 developed symptoms in the following days, mainly fever and respiratory distress, thus were considered pre-symptomatic (7.7 %), and 20 (21.9 %) remained asymptomatic. Mann Whitney U test was performed in order to evaluate the difference in the viral loads among asymptomatic, symptomatic and pre-symptomatic patients. Results showed that viral load median value was higher in the asymptomatic (5.79 × 10^4^) than in the pre-symptomatic group (5.33 × 10^2^) (*p* = 0.022) (Table 4), while no difference was revealed for *age* and *sex* variables.

**Table 4:** Evaluation of viral load, sex and age variables among asymptomatic and pre-symptomatic patients

	Total	Viral load (copies/mL)			Sex		Age	
		Mean	Median	SD	Male	Female	Mean	Median
**Overall**	27	3.99E+07	1.14E+07	7.03E+07	12	15	68	74
**Asymptomatic**	20	1.75E+05	**5.79E+04**	2.6E+05	9	11	66	74
**Pre-symptomatic**	7	1.19E+04	**5.33E+02**	2.01E+04	3	4	74	78

We also evaluated the presence of comorbidities among the SARS-CoV-2 confirmed cases. Pre-symptomatic individuals showed the presence of comorbidities in 5 cases (71.4 %), 3 diabetes and hypertension, 1 chronic obstructive pulmonary disease and 1 intracranial meningioma, that died later. In the symptomatic group comorbidities were found in the 68.7 % (*n* = 44), where hypertension was the most common (*n* = 29; 65.9 %) followed by diabetes (*n* = 14; 31.8 %) and severe respiratory disorders (*n* = 9; 20.4 %), finally, the 20 % (*n* = 4) of the patients that remained asymptomatic showed concomitant diseases, mostly diabetes and hypertension (extended data in the Additional file). We observed that the presence of comorbidities was an important risk factor for the presence and development of symptoms, indeed symptomatic and pre-symptomatic were found to be 8.9 folds more at risk of showing a clinical course of the disease (*p* < 0.0001) (Table 5).

**Table 5:** Correlation between comorbidities, the presence/development and the absence of symptoms

	Total	Presence of comorbidity		X^**2**^	***p*** value	OR	OR 95% CI
		n	%				
***Symptom presence and development***	71	49	69	**15.41**	**<0.0001**	**8.9**	**2.6 -29.7**
***Symptom absence***	20	4	20				
***Total***	91	53	58.2				

Long-term follow-up data, concerning serological tests, re-infections, long-term symptomatology and decease were collected. Qualitative serological tests for the detection of IgG antibodies were conducted by the health care workers within the two facilities. Among symptomatic, asymptomatic and pre-symptomatic patients, 29 (45.3 %), 7 (35 %) and 3 (42.8 %) were found positive, respectively. Moreover, 3 (3.3 %) patients re-infected at 38, 58 and 181 days post-infection and 3 patients (3.3 %) showed long-term asthenia and shortness of breath (extended data in the Additional file). Finally, 12 people died, 8 female (66.6 %) and 4 male (33.3 %).

## Discussion

In this study we will try to elucidate the spread of infection in two nursing facilities during the Italian lockdown first period, occurred in late March 2020. Notably, because of the lockdown, health care workers were not able to move along and the use of Personal Protective Equipment (PPE) was mandatory. We could suppose that the virus, after the infection establishment inside these facilities, circulated among health care workers and patients and caused a high infection rate (50.8 %). Risk factors that can create higher susceptibility to SARS-CoV-2 infection, such as age, sex, viral load and concomitant diseases, are frequently discussed. Elderly people are supposed to be particularly susceptible to the infection, especially in over 65 year old [7, 8, 11–22], indeed, our findings showed this category was 3.93 folds more susceptible to the infection than under 65 year old patients, moreover, tested positive cases had a mean and median age values​​ higher than tested negative (*p* = 0.001). Sex was also found to be a risk factor, thus female showed 2.86 folds higher infection rates of SARS-CoV-2 than male, corroborating other authors’ findings [6–8, 10–22]. Elderly and female groups are confirmed to have higher frequencies of SARS-CoV-2 infection, indeed age and sex can be considered important risk factors, as already described by other authors [5–22], nevertheless some studies have reported different results on COVID-19 sex-related attack rates. High viral load is also considered another relevant factor for the transmission of the pathogen but its role on the clinical course of the disease is still unclear. Some authors have described various scenarios in which the higher viral loads were observed in symptomatic elderly people [6–14, 18–22] instead of other cases where in asymptomatic individuals higher viral loads were found [5], as well as upper respiratory traits persistent positivity [21].

The spreading difference of the infection among asymptomatic and symptomatic individuals is reported in several papers but how they could spread the disease has not been definitively clarified [23], nevertheless, sporadic transmissions from non-symptomatic people are described [8, 11–25], that makes their involvement an important issue to take into account in the circulation of the virus. Some surveys have hypothesized the rule of super-spreaders during infection outbreaks. Kumar and colleagues identified clinical and social characteristics of these super-spreaders, such as heavy dose of infection and high viral shedding and may have more severe cough, thereby they are more likely able to spread the infection. Social habits, which increase the transmission rates, are individuated in travelling to many places, public gathering, working in crowded places or in confined spaces and hospital staff is included in the list of possible super spreaders [26]. Indeed, health care institutes have been identified as the fourth most considerable causes of disease spreading [22] thus it should also be supposed that high density settings are at viral circulation high risk [27]. Our findings revealed that a non-negligible percentage of positive individuals (29.7 %) showed an asymptomatic clinical course, moreover, median viral load resulted statistically significant higher when compared to symptomatic ones, corroborating other authors’ findings [5]. It is strongly controversial the asymptomatic stage evolving in symptomatic, as well as the viral loads in both classes. A distinction between asymptomatic and pre-symptomatic stages can currently only be made retrospectively, after the occurrence or non-occurrence of clinical symptoms [28]. Indeed, the 21.9 % of the non-symptomatic cases at swab sampling time, never developed symptoms, thus as already reported a range from 9.2 to 69 % of confirmed SARS-CoV-2 cases remain asymptomatic [8, 11–29]. Furthermore, when viral loads of asymptomatic and pre-symptomatic patients were evaluated, median value was higher in the asymptomatic (5.79 × 10^4^) than in the pre-symptomatic group (5.33 × 10^2^) (*p* = 0.022), thus, these observations could suggest that viral loads are not related to the severity of the disease.

Concomitant diseases are also discussed to create higher susceptibility to the infection and development of COVID-19 related symptoms. Patients with cardiac diseases, hypertension and diabetes are at higher risk for severe infection [27–30]. Presence and development of symptoms seemed to be comfortably related to the presence of such comorbidities when compared to non-symptomatic cases (OR = 8.9; *p* < 0.0001).

## Conclusions

During the first phase of the Italian pandemic caused by SARS-CoV-2 (March-May 2020), on WHO recommendation, oro-pharyngeal swabs were performed only in symptomatic subjects with severe acute respiratory syndrome or in pauci-symptomatic patients with epidemiological correlations to other infected. To date, Italian surveillance continues to be somehow on a voluntary basis, but due to the results of many international papers, the role of asymptomatic people, showing high viral loads, can be no longer ignored, mostly in relation to the spread of the disease, especially in an environment, which certainly has not only important intrinsic (age, sex, concomitant diseases) but also extrinsic factors such as high population density and close contacts. It is therefore necessary to extend epidemiological surveillance to wider cohorts. Certainly, nursing facilities and hospitals are of primary importance as well as schools, gyms, and all places where necessary gatherings occur. Tracking, detection and timing of asymptomatic case could reduce the cumulative number of disease cases. Public health measures should be improved to address this challenge.

## Supplementary Information

**Additional file 1.**

## Data Availability

The dataset supporting the conclusions of this article is included within the article and its additional file.

## Abbreviations

SARS-CoV-2: Severe Acute Respiratory Syndrome- Coronavirus-2
COVID-19: Coronavirus disease 19
WHO: World Health Organization
ECDC: European Centre of Disease Control and Prevention
IZSM: Istituto Zooprofilattico Sperimentale del Mezzogiorno
RT-qPCR: Reverse transcriptase quantitative polymerase reaction
BLS: Biosafety Level
UTM: Universal Viral Transport Medium
UNG: Uracil-DNA glycosylase
PPE: Personal Protective Equipment
OR: Odds ratio
CI: Confidence Intervals
SD: Standard Deviation
